# Real‐world data for the renal safety of abemaciclib combined with bisphosphonate in HR+/HER2− advanced breast cancer

**DOI:** 10.1111/1759-7714.14715

**Published:** 2022-11-09

**Authors:** Chunfang Hao, Xuedong Bai, Jie Zhang, Wenjing Meng, Zhongsheng Tong

**Affiliations:** ^1^ Present address: Department of Breast Oncology, Tianjin Medical University Cancer Institute & Hospital, National Clinical Research Center for Cancer, Key Laboratory of Cancer Prevention and Therapy Tianjin's Clinical Research Center for Cancer Tianjin China; ^2^ Department of Geriatrics Hebei General Hospital Shijiazhuang China; ^3^ Department of Breast Oncology, Tianjin Cancer Hospital Airport Hospital, Tianjin Medical University Cancer Institute & Hospital, National Clinical Research Center for Cancer, Key Laboratory of Cancer Prevention and Therapy Tianjin's Clinical Research Center for Cancer Tianjin China

**Keywords:** abemaciclib, bisphosphonate, breast cancer, HR+/HER2−, renal safety

## Abstract

**Objective:**

Our study evaluated the renal safety of abemaciclib plus endocrine therapy (ET) with bisphosphonate as a treatment option for hormone receptor positive, human epidermal growth factor receptor 2 (HER‐2) negative (HR+/HER2−) advanced breast cancer (ABC), especially with bone metastasis.

**Methods:**

Data were collected from HR+/HER2− ABC patients who received abemaciclib with ET between March 2021 and May 2022 in a single medical center in China. We performed an analysis of the change in serum creatine (Cr) and creatine clearance (CrCl), time to first abnormal Cr value, and Common Terminology Criteria for Adverse Events grade of increased creatinine.

**Results:**

A total of 210 patients were included in the final analysis, with a median age of 56 years and a median weight of 65 kg. Any grade laboratory‐assessing increased Cr occurred in 87.1% of patients, while CrCl rarely went down to 30 ml/min. Associations between start dose with grade of increased Cr and menopausal status with alert value, which is defined as creatinine clearance <30 ml/min, were indicated.

**Conclusion:**

This study shows that abemaciclib combined with bisphosphonate would be safe for renal function in HR+/HER2− ABC patients with bone metastases.

## Graphical abstract

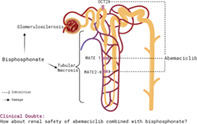

## INTRODUCTION

Breast cancer (BC) is the most commonly diagnosed cancer worldwide among women and the second most common cause of cancer death.^1^ Regarding the information published by the International Agency for Research on Cancer (IARC) of the World Health Organization (WHO), the morbidity of BC (19.9%) ranks first among cancers among Chinese women, and the mortality rate (9.9%) ranks fourth among all cancers in 2020.^2^ About 70% of breast cancers are hormone receptor positive (HR+) and human epidermal growth receptor negative (HER2−).^3^ Endocrine therapies (ETs) are the basis of treatment for HR+/HER2– advanced breast cancer (ABC), but de novo or acquired resistance to metastatic breast cancer remains an important clinical challenge.

Many molecular mechanisms can result in ET resistance, including dysregulation of the cyclin‐dependent kinase (CDK) 4/6‐ inhibitor of CDK4 (INK4) ‐retinoblastoma proteins (Rb) pathway. Abemaciclib, a small‐molecule inhibitor of CDK4 and CDK6, is approved by the Chinese National Medical Products Administration for use in combination with ET for the treatment of HR+/HER2− ABC based on data from MONARCH plus.^4^ Randomized clinical trials of abemaciclib plus ET have shown notable survival gains and consistent safety profiles. It should be noted that increased creatinine is the most common laboratory abnormality.

Bisphosphonates are widely used as valuable antiresorptive agents for the treatment of post‐menopausal osteoporosis (PMO), hypercalcemia of malignancy, and osteolytic bone metastases.^5^ However, the patterns of nephrotoxicity were reported toxic acute tubular necrosis (ATN)^6^, ^7^ and collapsing focal segmental glomerulosclerosis (FSGS).^8^, ^9^ Therefore, all bisphosphonates carry labeled “warnings” or a contraindication for use in patients with severe renal impairment (creatinine clearance <30 ml/min).^10^

Under some conditions, physicians may be suspitions whether the combination of abemaciclib and bisphosphonates in the treatment of HR+/HER‐ breast cancer patients with bone metastases may aggravate renal abnormalities. The aim of this analysis was to evaluate the safety of real‐world use of abemaciclib plus different ETs with or without bisphosphonates in HR+/HER2− ABC.

## METHODS

### Study design and population

This was a retrospective cohort study whose purpose was to evaluate the renal safety of abemaciclib‐containing therapy in the treatment of metastatic breast cancer (MBC) in a real‐world setting. Eligible patients aged 18–80 years with metastatic or locally advanced and unresectable BC who were treated between March 2021 and May 2022, and had received at least one dose of abemaciclib were identified and included in the study. No other inclusion or exclusion criteria were used. Common Terminology Criteria for Adverse Events (CTCAE) grade, severity, incidence, seriousness, and timing of increased creatinine were all assessed. Local laboratory assessments were conducted at baseline, the first day of each cycle, and approximately 30 days after discontinuation of therapy. Demographic and clinical data (i.e., osteoclast inhibitors and CrCls at different times) were extracted from the hospital information system. This noninterventional retrospective study was approved by the responsible ethics committee at Tianjin Medical University Cancer Institute.

### Endpoints

The first endpoint was the rate of increased creatinine of abemaciclib‐based treatment as assessed according to CTCAE version 5.0. The second endpoint consisted of time to the first abnormal Cr value, change in Cr and CrCl, and the rate of alert value.

### Statistical analysis

Data were summarized using descriptive statistics. Categorical variables were summarized by number and percentage. Summary tables (descriptive statistics and/or frequency tables) were provided for all variables, as appropriate. Continuous variables were summarized using *n* (number of subjects with available data), median, and range (minimum and maximum) unless otherwise specified. Chi‐squared analysis and Fisher's exact test were used to determine whether there was a significant association between alert value/grade of increased creatinine and the following factors: age, weight, start dose, bone metastasis status, menopausal status, dose modification, ETs, osteoclast inhibitors, and baseline of Cr/CrCl. An arbitrary level of 5% statistical significance (two‐tailed) was used. Baseline characteristics associated with alert value and grade of increased creatinine were explored by univariable/multivariable logistic regression. R version 4.1.3 was used to perform the statistical analyses.

## RESULTS

### Patient characteristics

In a cohort of 133 postmenopausal women, 210 HR+/HER2− ABC patients were identified and included in the analysis. The median age was 56 years (range 31–79) and the median weight was 65 kg(range 44.5–90.0). One hundred and eighty patients (85.7%) had received the full recommended dose of abemaciclib at the beginning of treatment and 33 (15.7%) had performed dose reduction. Seventy‐eight patients (37.1%) had been diagnosed with bone metastasis, of which 69 (32.9%) had taken bisphosphonates. The most common endocrine therapy combined with abemaciclib was aromatase inhibitors (105 patients, 50.0%) followed by fulvestrant (96 patients, 45.7%) and tamoxifen (TAM) (six patients, 2.9%). The patients' demographic and clinical characteristics, as well as the value of Cr and CrCl at baseline, are presented in Table 1.

**TABLE 1:** Patient demographic and treatment characteristics

All patients (*n* = 210)					
Age (years)	Median (range)	56 (31–79)	Type of endocrine therapy		
Weight (kg)	Median (range)	65 (44.5–90.0)		AI	105 (50%)
Menopausal status				Fulvestrant	96 (45.7%)
	Premenopausal	77 (36.7%)		TAM	6 (2.9%)
	Postmenopausal	133 (63.3%)		NA	3 (1.4%)
Bone metastases status			Start dose of abemaciclib (mg/bid)		
	Yes	78 (37.1%)		150	180 (85.7%)
	No	132 (62.9%)		100	27 (12.9%)
Osteoclast inhibitors				50	3 (1.4%)
	Bisphosphonate	66 (31.4%)	Dose modifications		
	Denosumab	6 (2.9%)		Yes	33 (15.7%)
	Bisphosphonate/denosumab	3 (1.4%)		No	165 (78.6%)
	None	132 (62.9%)	Cr at baseline	Median (range)	59.5 (34–95)
	NA	3 (1.4%)	CrCl at baseline	Median (range)	90.79 (32.05–211.09)

*Abbreviations*: AI, aromatase; Cr, creatine; CrCl, creatine clearance; NA, not access; TAM, tamoxifen.

### Renal safety

A total of 183 patients (87.1%) experienced any grade of increased creatinine by laboratory assessment. The rates of grades 1 and 2 were 57.1% and 29.5%, respectively. No grade ≥3 events of increased creatinine were reported. The median time to onset (first abnormal Cr from the treatment) was 10.1 weeks (range 0–38.4). The mean change in Cr was 24.7 μmol/L and in CrCl was 27.9 ml/min. Chi‐squared analysis and Fisher's exact test showed a statistically significant association in patients with the full recommended start dose of abemaciclib (*p* = 0.04) (Table 2) in postmenopausal patients and a higher grade of increased creatinine (*p* = 0.00) (Table 3).

**TABLE 2:** Chi‐squared analysis and Fisher's exact test for alert value

		CrCl ≥ 30 ml/min	CrCl < 30 ml/min	*p*			CrCl ≥ 30 ml/min	CrCl < 30	*p*
Age (years)				1.00	Type of endocrine therapy				0.21
	<60	125	4			AI	103	2	
	≥60	79	2			Fulvestrant	93	3	
Weight (kg)						TAM	5	1	
	<68.5	137	6	0.18		NA	3	0	
	≥68.5	67	0		Start dose of abemaciclib (mg/bid)^a^				0.04
Menopausal status				0.09		150	177	3	
	Premenopausal	77	0			100	24	3	
	Postmenopausal	127	6			50	3	0	
Bone metastases status				0.09	Dose modifications				0.05
	Yes	78	0			Yes	33	0	
	No	126	6			No	161	4	
Osteoclast inhibitors				0.46		NA	10	2	
	Bisphosphonate	66	0						
	Denosumab	6	0						
	Bisphosphonate/denosumab	3	0						
	None	126	0						
	NA	3	6						

*Abbreviations*: AI, aromatase inhibitors; bid, bis in die; Cr, creatine; CrCl, creatine clearance; NA, not access; TAM, tamoxifen.

^a^ The difference between groups is significant.

**TABLE 3:** Chi‐squared analysis and Fisher's exact test for grade of increased creatinine

		Grade 1	Grade 2	*p*			Grade 1	Grade 2	*p*
Age (years)				0.81	Type of endocrine therapy				0.44
	<60	82	38			AI	65	28	
	≥60	48	24			Fulvestrant	59	31	
Weight (kg)						TAM	3	3	
	<68.5	91	37	0.16		NA	3	0	
	≥68.5	39	25		Start dose of abemaciclib (mg/bid)				0.33
Menopausal status^a^				0.00		150	115	53	
	Premenopausal	56	13			100	12	9	
	Postmenopausal	74	49			50	3	0	
Bone metastases status				0.18	Dose modifications				0.18
	Yes	53	19			Yes	24	6	
	No	77	43			No	100	50	
Osteoclast inhibitors				0.28		NA	6	6	
	Bisphosphonate	42	18						
	Denosumab	6	0						
	Bisphosphonate/denosumab	2	1						
	None	77	43						
	NA	3	0						

*Abbreviations*: AI, aromatase inhibitors; bid, bis in die; NA, not access; TAM, tamoxifen.

^a^ The difference between groups is significant.

## DISCUSSION

In recent years, four CDK4/6 inhibitors have been approved worldwide for the treatment of HR+/HER2− ABC. Several randomized clinical trials (palbociclib, the PALOMA studies; ribociclib, the MONALEESA studies; abemaciclib, the MONARCH studies; dalpiciclib, the DAWNA studies) have proved that they are generally safe and well tolerated, and, importantly, improved both progression free survial (PFS) and overall survival (OS) in HR+/HER2− MBC.^11^, ^12^, ^13^, ^14^, ^15^, ^16^, ^17^, ^18^, ^19^, ^20^, ^21^, ^22^, ^23^ The differences among these CDK4/6 inhibitors include, but are not limited to, administration schedules (ribociclib, palbociclib, and dalpiciclib: once daily 3 weeks on, 1 week off; abemaciclib: twice daily) and varying toxic profiles.

In previous studies, abemaciclib exhibited more marked serum creatinine abnormalities than other CDK4/6 inhibitors. Increased creatinine was observed in 98.3% of abemaciclib‐treated patients in MONARCH 2 and 3^23^ (434 patients in the MONARCH 2 abemaciclib arm; 219 patients in the MONARCH 2 placebo arm; 314 patients in the MONARCH 3 abemaciclib arm; 156 patients in the MONARCH 3 placebo arm). A similar incidence (87.1%) was observed in most cases in our study (Table 4). Creatinine elevation is known to occur with abemaciclib because abemaciclib and its major active metabolites inhibit the renal organic cation transporter (OCT) 2, multidrug and toxin extrusion (MATE) 1, and MATE2‐K at concentrations achievable at the approved recommended dosage.^24^ Abemaciclib and its major metabolites at clinically relevant concentrations do not inhibit the hepatic uptake transporters organic cation transporter 1 (OCT1), organic anion transporting polypeptide (OATP)1B1, and OATP1B3 or the renal uptake transporters organic anion transporter (OAT)1 and OAT3.

**TABLE 4:** Changes in creatinine in MONARCH 2, MONARCH 3, and this study

Characteristics	MONARCH 2		MONARCH 3		Our study	
	Abemaciclib + fulvestrant (*n* = 441)	Placebo + fulvestrant (*n* = 223)	Abemaciclib + NSAI (*n* = 327)	Placebo + NSAI (*n* = 161)	Abemaciclib + fulvestrant (*n* = 96)	Placebo + AI (*n* = 105)
Creatinine increased, by laboratory assessment						
Any grade	427 (98.4%)	161 (73.5%)	308 (98.1%)	131 (84.0%)	90 (93.8%)	93 (88.6%)
Grade 1	231 (53.2%)	154 (70.3%)	135 (43.0%)	124 (79.5%)	59 (61.5%)	65 (61.9%)
Grade 2	191 (44.0%)	7 (3.2%)	166 (52.9%)	7 (4.5%)	31 (32.3%)	28 (26.7%)
Grade 3	5 (1.2%)	0 (0)	7 (2.2%)	0 (0)	0 (0)	0 (0)
Grade 4	0 (0)	0 (0)	0 (0)	0 (0)	0 (0)	0 (0)

Bones are one of the most common metastatic sites for breast cancer and lung cancer. Bone metastases can significantly increase mortality and decrease the quality of life of cancer patients through pain, pathologic fractures, spinal cord compression, and hypercalcemia of malignancy. Osteoclast inhibitors (also called antiresorptive agents), such as bisphosphonates and denosumab, significantly reduce the frequency of and delay the time to onset of skeletal‐related events in patients with bone metastases. Recent experiments have shown that bisphosphonates inhibit farnesyl diphosphate in a human proximal tubular cell line and that this step may be followed by decreased levels of prenylated proteins and later cytotoxicity, an observation that strongly supports the concept that the therapeutic effect of bisphosphonates on osteoclasts may have an identical mechanism to the toxic effect on the kidney.^5^ However, none of the 69 patients taking bisphosphonates exceeded alert value in this real‐world study, and it supports the combination of abemaciclib with bisphosphonates used in HR+/HER2− ABC with bone metastasis.

There are several potential limitations to this retrospective single‐center cohort study. First, the results should be interpreted cautiously, and the selection bias should not be ignored due to its retrospective nature. Second, the small sample size may have led to chance findings or masked correlations, thus affecting the results; meanwhile, the collection of data, for example survival information, was incomplete. Finally, the follow‐up time was not long enough to determine whether abemaciclib plus ET with/without bisphosphonates would produce cumulative nephrotoxicity.

## CONCLUSION

In general, the results of this study indicate that the toxicity profiles were similar to the prior conducted clinical trials. An implication of this is the possibility that abemaciclib plus ET with/without bisphosphonates has no influence with renal function in HR+/HER2− ABC.

## AUTHOR CONTRIBUTIONS

Chunfang Hao, Xuedong Bai, Jie Zhang, Wenjing Meng, and Zhongsheng Tong conceived and designed the study. Chunfang Hao, Xuedong Bai, Jie Zhang, and Wenjing Meng analyzed the data. Chunfang Hao, Xuedong Bai and Jie Zhang wrote the paper. Zhongsheng Tong provided administrative support and gave final approval of the version to be published.
